# Alternative splicing of KLF4 in myeloid cells: implications for cellular plasticity and trained immunity in cancer and inflammatory disease

**DOI:** 10.3389/fimmu.2025.1585528

**Published:** 2025-06-09

**Authors:** Amanda Rosewell Shaw, Attoria Bennett, Daping Fan, Walden Ai

**Affiliations:** ^1^ Department of Biology, Benedict College, Columbia, SC, United States; ^2^ Center for Cell and Gene Therapy, Baylor College of Medicine, Texas Children’s Hospital, Houston Methodist Hospital, Houston, TX, United States; ^3^ Department of Cell Biology and Anatomy, University of South Carolina School of Medicine, Columbia, SC, United States

**Keywords:** Krüppel-like factor 4 (KLF4), AS, cellular plasticity, cancer stem cells, trained immunity, innate immunity, myeloid-derived suppressor cells (MDSCs), epigenetics

## Abstract

The role of transcription factor Krüppel-like factor 4 (KLF4) in the modulation of myeloid cells is well known. KLF4 is involved in the differentiation and polarization of monocytes and macrophages as part of the immune response after infection, in wound healing, and in cancer. In addition, KLF4 is essential in stem cell reprogramming and the phenomenon of trained immunity – a form of innate immune memory marked by epigenetic and metabolic reprogramming. A novel and underexplored dimension of KLF4 biology lies in its alternative splicing (AS), which generates distinct isoforms that may drive the transcription factor’s functions, depending on specific cellular environments, disease states, or signaling programs. This review presents current knowledge of KLF4 splicing in myeloid cells and explores novel connections for how KLF4 isoform diversity may contribute to cellular plasticity and differential immune responses of myeloid cells across physiological and pathological conditions.

## 1 Introduction

The discovery that innate immune cells can exhibit memory-like behavior, once thought exclusive to the adaptive immune system, has reshaped fundamental assumptions in immunology (1, 2). This phenomenon, known as trained immunity, is driven not by genetic rearrangement of lymphoid cells but by transcriptional and epigenetic reprogramming of myeloid cells (3). While the transcription factor Krüppel-like factor 4 (KLF4) is known to regulate these processes (4, 5), an emerging and underappreciated layer of complexity lies in its alternative splicing (AS) (6). This review explores how distinct KLF4 isoforms, generated through context-specific splicing events, may act as molecular switches modulating myeloid plasticity and trained immunity with far-reaching implications for inflammation, cancer, and regenerative medicine.

### 1.1 Krüppel-like factor family and KLF4 biology

Myeloid cells are a heterogeneous group of hematopoietic cells that play essential roles in innate and adaptive immune responses. Myeloid-progenitor cells are produced from hematopoietic stem cells (HSCs) in the bone marrow and then differentiate through complex gene-regulatory mechanisms into myeloid-linage cells such as monocytes, macrophages, dendritic cells, and neutrophils, among others (7). These cells can directly detect pathogens and eliminate them in a non-specific manner, but also play key roles in initiating the adaptive arm of the immune system’s response.

The Krüppel-like factor (KLF) family is a highly conserved group of transcription factors containing zinc finger domains, allowing them to bind GC-rich regions of promoter and enhancer elements of the genes they regulate. There are seventeen known mammalian KLFs, essential in a wide range of biological mechanisms, including proliferation, differentiation, development, cellular responses, and normal tissue homeostasis (4, 5). KLFs are known to have essential roles in hematopoiesis, with KLF4 identified as having a pivotal role in myelopoiesis and a key factor in monocyte differentiation (8).

### 1.2 KLF4 in stemness and reprogramming

KLF4 regulates many cellular processes and can act as a transcriptional activator or repressor of many target genes. KLF4 is essential for survival, as KLF4-null mice die shortly after birth due to dehydration from the loss of the barrier function of the skin (9). The interesting dichotomy of KLF4 is that it plays essential roles in cellular differentiation and cell cycle regulation, such that dysregulation results in impaired terminal differentiation or uncontrolled cellular proliferation (6). Together with OCT4, SOX2, and MYC, KLF4 makes up the Yamanaka factors (10), which allow for the direct reprogramming of somatic cells to a pluripotent state, demonstrating KLF4’s importance for cellular plasticity. To that end, KLF4 is critical in the differentiation, polarization, and function of myeloid cells, including monocyte responses to environmental factors (8), thereby impacting innate immune memory.

### 1.3 KLF4 is implicated in trained immunity

Traditionally, the immune system has been divided into an innate arm, composed of cells that are rapidly activated by non-specific pathogen- and damage-associated patterns, and an adaptive arm, which recognizes specific targets of pathogens and forms a slow response but long-lasting immune memory, allowing for rapid response to repeated stimuli. Until recently, immune memory was thought to be solely a function of adaptive immune responses. Recent work has challenged this dogma and demonstrated that innate immune cells could mediate adaptive characteristics and has since been termed ‘trained immunity’ (1, 2, 11). Trained immunity is mediated by the epigenetic reprogramming of transcriptional pathways in myeloid cells, in contrast to genetic recombination in adaptive lymphocytic immune cells (3). The KLF4 master regulator has been implicated in this transcriptional reprogramming of myeloid cells to induce trained immunity in response to infection (12).

### 1.4 AS of KLF4 produces functionally distinct isoforms

**Figure 1:**
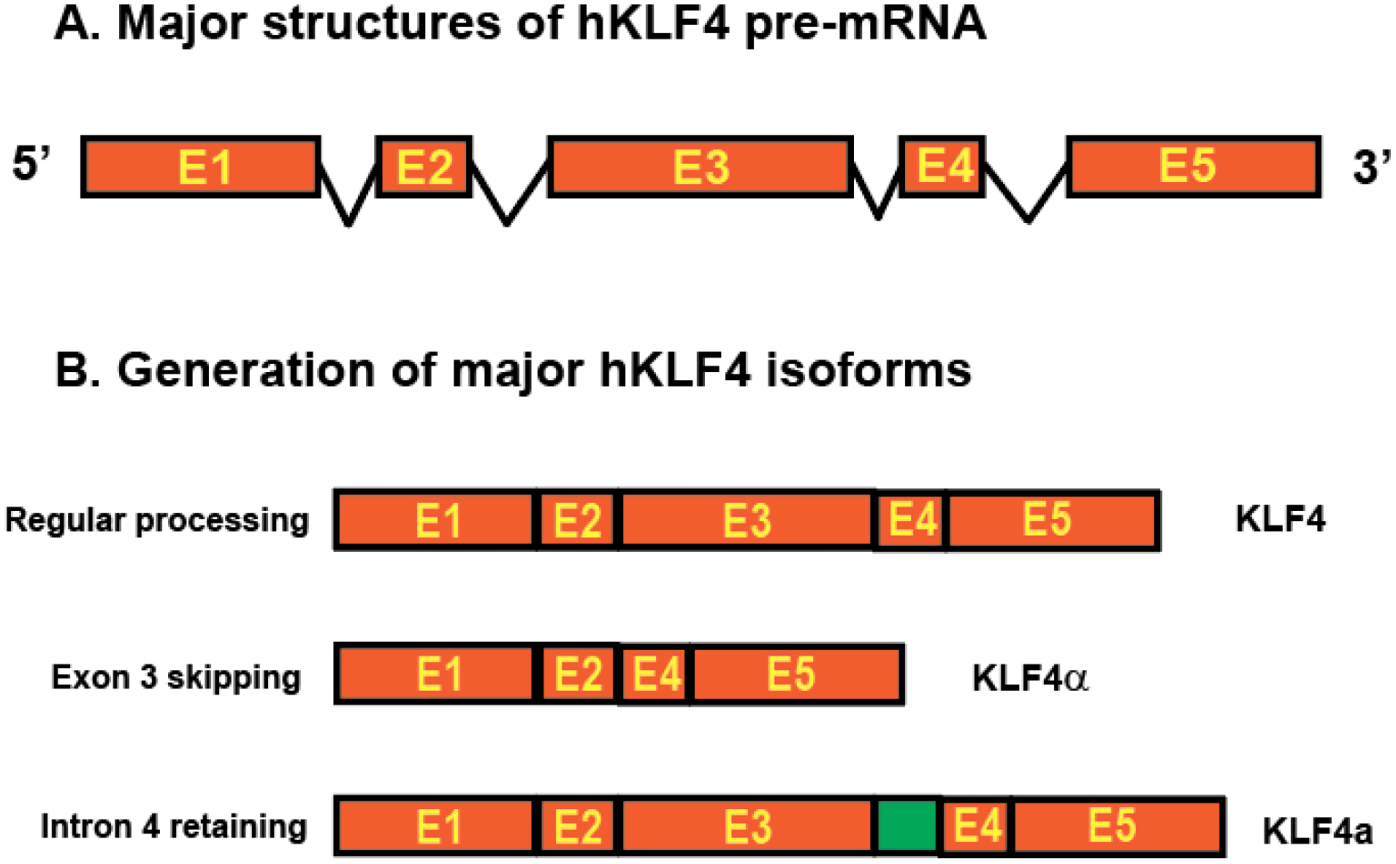
Structures of human KLF4 gene and major isoforms due to AS. **(A)** KLF4 gene structure. The orange boxes represent five exons and the lines between orange boxes represent introns. **(B)** AS of KLF4 pre-mRNA generates three major isoforms including the full length KLF4, shorter KLF4α with exon 3 skipping, and longer KLF4a with intron retained between exon 3 and exon 4 (green box).

Eukaryotic gene regulation occurs at multiple levels, including transcription of RNA from DNA, processing of primary transcripts to mature mRNA, translocation of mRNA from the nucleus to the cytoplasm, and translation of those mRNAs into proteins. Different mRNA transcripts from one single protein-coding gene have been identified due to the advancement of long-read sequencing technology. These RNA transcripts are produced through AS of the pre-mRNA of the protein-coding gene, which effectively diversifies genome complexity (13). AS of KLF4 pre-mRNA has been reported in pancreatic cancer, generating five different intron-skipping isoforms (14). One of the isoforms, KLF4α, which lacks the KLF4 exon 3 sequences, antagonizes the function of full-length KLF4 in breast cancer and pancreatic cancer (14, 15). In addition to intron-skipping isoforms, an intron-retaining isoform KLF4a, retaining a 102-bp in-frame intronic region between exons 3 and 4 of the coding sequences of the human KLF4 gene, was identified in immune cells (16, 17). The unique function of the KLF4a isoform is unknown (**Figure 1**). It is proposed that AS of KLF4 pre-mRNA mediates its context-dependent functions (6).

Despite growing recognition of KLF4’s importance in immune regulation and myeloid reprogramming, little is known about the specific roles of its alternatively spliced isoforms. Key questions remain unanswered: How are these isoforms differentially expressed across tissues and physiological conditions? Do they exert opposing or synergistic functions, and in what contexts? How do they contribute to cellular plasticity in health and pathological states? These gaps are significant because different KLF4 isoforms may underlie its paradoxical roles as both tumor suppressor and oncogene, as well as its dual influence on inflammatory and anti-inflammatory myeloid phenotypes. Clarifying these functional differences is essential not only for mechanistic understanding but also for resolving current contradictions in the field and improving the specificity of therapeutic strategies that target KLF4.

Here, this review will explore the role of KLF4 in myeloid cell biology to provide the foundation of its activities in monocyte and macrophage differentiation, the signaling pathways involved, and the epigenetic modification mediated by KLF4. Moreover, we will present the implications of KLF4 regulation of myeloid cells in infection, wound healing, and cancer, and delve into the potential of KLF4 as a regulator in trained immunity.

Finally, we will propose how AS of KLF4 pre-mRNA may influence the function of myeloid cells. Full-length KLF4 is known to influence chromatin accessibility by binding to methylated enhancer elements and recruiting histone-modifying enzymes and remodeling complexes, shaping transcriptional programs (18). However, it remains unclear how alternatively spliced isoforms alter these interactions or rewire epigenetic landscapes. Given that the KLF4α lacks the DNA binding domain, this isoform may have diminished capacity for epigenetic reprogramming.

As trained immunity is driven by sustained changes in chromatin accessibility, elucidating how specific KLF4 isoforms participate in or disrupt these epigenetic programs is essential to understanding their role in innate immune memory and cellular plasticity. Understanding the connection of KLF4 isoforms to cellular plasticity and its role in mediating immunological memory in innate immune cells may have significant therapeutic implications in immune-related disorders and cancer.

## 2 KLF4 in myeloid cell biology

KLF4’s role in the myeloid lineage is essential for numerous functions, such as differentiation, polarization, and responses to biological stressors, including immune responses to infection, wound healing, and cancer. Myeloid cells are environmental sensors, and their plasticity is regulated by their microenvironment (19). For example, within the tumor microenvironment, factors such as hypoxia, ER stress, exosomes, and tumor-derived signaling molecules like granulocyte-macrophage colony-stimulating factor (GM-CSF) and vascular endothelial growth factor (VEGF) contribute to myeloid plasticity (20). KLF4 is crucial for monocyte differentiation from myeloid progenitor cells (8). Monocytes are phagocytic cells of innate immunity, engulfing pathogens and, in turn, secreting signaling molecules to initiate an inflammatory cascade and attract other immune cells to the site of infection or damage. KLF4 is highly expressed in monocytes and binds to the monocyte-specific CD14 promoter. It was found to be expressed in a stage-specific manner during myelopoiesis as knockout of KLF4 impeded the formation of mature monocytes and increased the population of granulocytes (8).

In addition to its essential role in monocyte development, KLF4 regulates the differentiation of monocytes into macrophages and modulates the transcriptional regulation of macrophage polarization, a process by which macrophages display their plasticity to adapt and execute different roles in response to environmental signals such as cytokine exposure (21). Macrophages are essential players in immune responses and exhibit functional plasticity, broadly classified into M1 (pro-inflammatory) and M2 (anti-inflammatory) phenotypes in which macrophages adopt different functional programs in response to signals from their surroundings. However, this binary model is now considered to be oversimplified. Recent studies suggest that macrophages can show a plethora of phenotypes, with plasticity allowing them to adopt functional characteristics of both M1, proinflammatory, and M2, anti-inflammatory mediators, based on the microenvironment (22). The mechanisms of this cellular plasticity are an ongoing area of study.

M1 macrophages are critical for the elimination of pathogens. However, their activity must be regulated so as not to result in chronic inflammation. KLF4 was found to be differentially expressed in macrophages after exposure to M1 or M2 stimuli. After bacterial endotoxin lipopolysaccharide (LPS) treatment (M1 stimulant), KLF4 is transiently increased, followed by significant downregulation (22). M2 macrophages are the counterbalance to M1s as they are involved in the resolution of inflammation. Macrophages stimulated with M2 factors IL-4 or IL-13 were found to have a sustained increase in KLF4 expression (22). KLF4 promotes the differentiation of macrophages towards the M2 phenotype by elevating levels of anti-inflammatory cytokines and factors that support tissue repair.

KLF4 suppresses M1 macrophage activation, at least in part, by sequestering coactivators necessary for NF-κB activation (22). For example, in the heart, KLF4 suppresses M1 polarization in tissue-resident macrophages, thereby reducing cardiac inflammation and atherosclerosis (23). However, studies suggest that KLF4 may also promote M1 polarization via activation of the STAT1 pathway. Overexpression of KLF4 in macrophages increased M1 markers TNF-α, IL-6, and IL-1β (24). Epigenetic modifications have also been shown to influence KLF4 activity. Posttranscriptional SUMOylation of KLF4 modulates its transcriptional activity and de-SUMOylation of KLF4 by the SENP1 protease enhances proinflammatory gene expression after LPS stimulation (25).

Conversely, upon stimulation with M2-associated cytokines such as IL-4, KLF4 expression is upregulated in macrophages and, through STAT6 signaling, induces M2-specific genes, including those involved with lipid metabolism, and the subsequent phenotypic polarization toward an anti-inflammatory M2 phenotype. KLF4 also suppresses the glycolytic pathway in M2 macrophages. KLF4-deficient macrophages have increased glucose uptake, indicating increased glycolysis, a feature of M1 macrophages (22). Thus, KLF4 regulates the metabolic reprogramming of macrophages and response to cytokines and environmental signals to mediate macrophage polarization.

The opposing effects of KLF4 in M1 versus M2 macrophage polarization (**Table 1**) raise the possibility that AS of KLF4 and the resulting distinct isoforms may mediate these divergent outcomes. For example, the full-length nuclear KLF4 may promote M2 polarization through transcriptional activation of anti-inflammatory genes, while cytoplasmic isoforms such as KLF4α, which lack the DNA-binding domain, may fail to repress NF-κB therefore contributing to proinflammatory M1 phenotypes. Additionally, recent studies using single-cell analysis have revealed distinct monocyte subsets, including classical, intermediate, and non-classical, among others, with differential inflammatory potential (26). It is currently unknown whether KLF4 isoforms have a role in this monocyte heterogeneity.

**Table 1:** Summary of KLF4’s Role in M1 and M2 macrophage polarization.

Stimulus	Pathway	KLF4 Role	Outcome
LPS	TLR4 → NF-κB	Transient ↑	M1
	deSUMOylation by SENP1	Activates M1 gene programs	M1
High-dose LPS	Mitochondrial stress and NAD+ depletion	STAT1-mediated ↑	Exhausted M1
IL-4/IL-13	STAT6 activation, metabolic shift away from glycolysis	Sustained ↑	M2

→, indicates the upstream and downstream relationship in a signal transduction pathway. ↑, indicates the upregulation of KLF4.

Myeloid-derived suppressor cells (MDSCs) are a heterogeneous group of immature myeloid cells that act as suppressors of immune responses and proliferate during pathologic states such as infection, inflammation, and cancer. In cancer, MDSCs are robust inhibitors of anti-tumor immune responses of T- and NK-effector cells. Additionally, MDSCs are involved in tumor angiogenesis, tumor invasion, and the development of pre-metastatic niches to promote cancer metastasis (27). Studies have demonstrated that KLF4 promotes the differentiation of MDSCs into fibrocytes, which are involved in tissue repair and fibrosis (28, 29). This differentiation process is crucial in the tumor microenvironment, where MDSCs contribute to immunosuppression and tumor progression. Regulation of the plasticity of myeloid cells by KLF4 between MDSCs and fibrocytes was also shown in an inflammatory response in the airway of a mouse model (30).

Given KLF4’s established role in guiding the differentiation of MDSCs into fibrocytes, it is plausible that this transition is mediated by specific KLF4 isoforms rather than the full-length protein alone. However, the transcriptional circuits through which KLF4 promotes MDSC-to-fibrocyte differentiation remain poorly understood. This is particularly significant as MDSCs are known to undergo extensive epigenetic reprogramming in response to tumor-derived signals (31), suggesting that KLF4’s activity in this context is likely modulated by chromatin accessibility and histone modifications. Full-length KLF4 may initiate this transition through canonical transcriptional activation, while alternative isoforms such as KLF4a or KLF4α may modulate chromatin structure or interact with noncanonical cofactors to alter fibrocytic fate determination. The interplay between KLF4 isoforms and chromatin-modifying enzymes within MDSCs warrants deeper investigation and may uncover new mechanisms of immune plasticity relevant to wound repair, fibrosis, and tumor progression.

These observations of KLF4’s role in myeloid cell biology raise broader questions about how KLF4 isoforms regulate transcriptional outcomes in diverse myeloid contexts. Despite compelling evidence that KLF4 plays a regulatory role in myeloid plasticity, the specific downstream transcriptional targets, the functional relevance of distinct KLF4 isoforms, and their integration within the broader chromatin architecture remain largely undefined. Most studies to date have focused on full-length KLF4 without dissecting the contributions of AS-derived isoforms to myeloid fate decisions. Furthermore, how KLF4 activity is coordinated with other lineage-determining transcription factors remains unclear. Given that PU.1 is a master regulator of myeloid lineage specification and chromatin priming (32, 33), it is plausible that KLF4 functions within the PU.1 pathway in a cell-type- and isoform-specific manner. Elucidation of whether KLF4 collaborates with or counterbalances PU.1 or other master myeloid regulators, and how these interactions are modulated by specific isoforms and epigenetic mechanisms, will be essential for clarifying the transcriptional programs of myeloid plasticity. Future studies should aim to map the isoform-specific binding partners and chromatin landscapes associated with KLF4 activity to resolve these outstanding questions.

## 3 KLF4 in innate immune memory

### 3.1 Mechanistic basis of trained immunity

The traditional dogma of immunology breaks the immune system into two arms: innate and adaptive. The innate arm responds quickly to signals from pathogens, pathogen-associated molecular patterns (PAMPS), such as bacterial or viral proteins and nucleic acids, or molecules released from dead or damaged cells, damage-associated molecular patterns (DAMPS), such as adenosine triphosphate (ATP) or high mobility group box 1 (HMGB1) (34). These innate responses were long considered nonspecific and incapable of forming immune memory, a role traditionally reserved for the adaptive arm of the immune system, as these cells undergo genetic rearrangement, allowing them to adapt to specific target signals and the development of long-lasting memory responses. However, this canonical understanding of immune memory has been challenged in recent years by the revelation that innate immune cells have enhanced immunological responses to secondary challenges, which occur through epigenetic and metabolic reprogramming of progenitor cells (35). While not as prolonged as adaptive immune memory, which can last for decades, innate immune memory can produce enhanced responses to secondary stimulation for up to 1 year (36), which is defined as trained immunity. It can be induced by β-glucan, a component of fungal and yeast cell walls (37), and a recent study investigating β-glucan induction of angiogenesis in human umbilical vein endothelial cells (HUVECs) demonstrated enhanced myocyte enhancer factor 2 (MEF2) transcriptional activity, which resulted in increased KLF4 expression (38).

Innate immune memory is driven by durable epigenetic remodeling, including histone modifications and chromatin accessibility changes that prime myeloid cells for enhanced responses to secondary stimuli (39, 40). KLF4 is well-positioned within this axis, as it is known to interact with key chromatin modifiers such as p300/CBP (histone acetyltransferases) (41), histone deacetylases (HDACS) (42, 43), and components of the SWI/SNF chromatin remodeling complex (44, 45). These interactions are essential during induced pluripotent stem cell (iPSC) reprogramming, where KLF4 helps establish permissive chromatin states at lineage-defining loci (46). Given this precedent, it is plausible that KLF4 performs a similar function during trained immunity by recruiting these epigenetic modifiers to inflammatory gene enhancers, facilitating chromatin looping and deposition of activating marks such as H3K27ac (18). This model suggests that KLF4 not only initiates transcriptional programs in response to training stimuli like β-glucan but also stabilizes them through chromatin remodeling. In addition, KLF4 is itself epigenetically modulated, a potential mechanism as a context-dependent tumor suppressor (47), adding another layer of complexity to KLF4 in epigenetic regulation. Further investigation is needed to determine whether KLF4 isoforms differentially influence epigenetic alterations and how such variation may contribute to the persistence and specificity of innate immune memory.

### 3.2 KLF4 in alveolar macrophage training

A direct link between KLF4 and trained immunity has been demonstrated by Chakraborty et al. They demonstrated that during infection with *Pseudomonas aeruginosa*, tissue-resident alveolar macrophages undergo KLF4-mediated transcriptional reprogramming, which confers a pro-efferocytosis phenotype (the phagocytic removal of dead or apoptotic cells) by upregulating MERTK (12). When these trained alveolar macrophages are transferred into naïve mice, they confer protection and prevent severe disease. Importantly, KLF4 is essential for MERTK upregulation and phenotypic shift in these tissue-resident macrophages, as when KLF4 was depleted, efferocytosis of cellular debris was markedly reduced. Additionally, the number of alveolar macrophages was increased after repeated pathogen exposure in a trained immunity model. It was found that this increase was not due to enhanced proliferation but a reduction in apoptosis, providing a potential link between reduced apoptosis and trained immunity (12). KLF4 negatively regulates p53 expression (48), which may contribute to enhanced macrophage survival during repeated pathogen exposures.

### 3.3 KLF4 and monocyte exhaustion

It has also been demonstrated that KLF4 is upregulated in the development of exhausted memory monocytes (49). In this study, the repetitive challenge of primary mouse monocytes with high doses of LPS skews monocytes into the classically exhausted Ly6C^high^ population and depletes the homeostatic non-classical Ly6C^low^ population. This exhaustion mirrors the monocyte dysfunction seen in sepsis, where immune paralysis leads to secondary infections and poor long-term outcomes. This persistence of dysfunctional responsiveness mimics a maladaptive form of immune memory and has been linked to innate exhaustion. Mechanistically, high doses of LPS robustly activate STAT1, which is dependent on the TRAM adaptor of the TLR4 pathway, and KLF4 is directly regulated by STAT1 in this setting. In addition, in line with the involvement of KLF4 in metabolic pathways discussed earlier in this paper, the generation of exhausted memory monocytes is associated with a drastic depletion of NAD+, elevation of ROS, and compromise of mitochondrial respiration.

### 3.4 Extension to lymphoid cells: ILC1s and beyond

In addition to the concept of trained immunity in the myeloid lineage, recent studies have shown that innate immune cells of the lymphoid lineage, termed innate lymphoid cells (ILCs), which include conventional natural killer (NK) cells (50, 51) mediate long-term memory-like responses (52). A recent study from Cheng et al. demonstrates that RAR-related orphan receptor alpha (RORα) is essential for the memory function of ILC1 and that several transcription factors, including KLF4, were upregulated in ILC1s with RORα-knockout (53). Because KLF4 is known to inhibit the proliferation of CD8^+^ T-lymphocytes (54) KLF4 may be implicated in the suppression of ILCs. Together, these findings suggest that KLF4 may function as a molecular bridge between innate training and adaptive conditioning. Future studies should explore its role in the monocyte-to-dendritic cell transition and its capacity to shape T cell responses through modulation of antigen presentation and cytokine expression profiles.

As KLF4 is also a known regulator of metabolic processes of cells that are altered during trained immunity, the importance of this transcription factor in this complex form of immune memory should be further explored. It remains unresolved how KLF4 integrates with canonical trained immunity mediators such as H3K4me3, fumarate, or mevalonate pathways. Moreover, whether KLF4 itself is epigenetically regulated during immune priming, as in tumor development, or if it directly recruits chromatin-modifying complexes warrants further mechanistic investigation.

While current evidence implicates full-length KLF4 in the transcriptional reprogramming of myeloid cells during trained immunity, it remains unclear whether alternative isoforms such as KLF4α and KLF4a contribute to the differential effector functions observed in this process. These isoforms differ significantly in their structural domains—KLF4α lacks the nuclear localization signal and DNA-binding domain, while KLF4a retains an intronic sequence that may influence protein interactions or stability, raising the possibility that they modulate immune memory through noncanonical mechanisms. Their subcellular localization and binding profiles could affect the transcriptional landscape differently from full-length KLF4, potentially influencing cytokine production, metabolic remodeling, or survival pathways during innate memory formation. Future studies should aim to determine whether these isoforms are expressed or functionally engaged in established models of trained immunity, such as β-glucan-induced or LPS-trained monocytes, to clarify whether isoform-specific roles underlie the context-dependent features of KLF4 in innate immune adaptation.

## 4 AS of KLF4 pre-mRNA and its implication

AS converts a pre-mRNA molecule into several mature mRNAs that can be translated into different proteins (55, 56). It is one of three major processing steps in eukaryotic mRNA maturation, including the 5’ capping by adding methylated GTP to the first transcribed nucleotide, 3’ poly-A tailing, and intron removal by splicing. AS is a major contributor to both protein diversity and control of gene expression levels, and it is a highly regulated process. AS is regulated at three interdependent levels: (i) RNA, via cis-regulatory elements and trans-acting factors; (ii) transcription, where RNA Polymerase II influences splice site choice; and (iii) epigenetics, where chromatin modifications affect spliceosome recruitment and activity. There is an intertwined relationship between these three levels of regulation, and likely with reciprocal feedback (13). In addition, AS in the immune system and tumor cells links to the events that can lead to AS dysregulation in tumors (57).

KLF4 has multiple roles in physiology and pathophysiology (5, 58–60). AS of KLF4 pre-RNA has been reported in mice and humans. For example, different KLF4 mRNA species have been reported in testis (61) and embryonic stem cells (62), leading to the generation of various exon-skipping KLF4 isoforms in mice. In humans, six different KLF4 splicing isoforms are reported in breast cancer, including full-length KLF4 and five other intron-skipping isoforms. It is proposed that the context-dependent functions of KLF4 are likely due to the generation of different KLF4 isoforms by AS of KLF4 pre-mRNA (6, 63) (**Table 2**). This is supported by an observation that the KLF4α isoform lacking the KLF4 exon 3 sequences antagonizes the function of full-length KLF4 in breast cancer and pancreatic cancer (14, 15). Lacking exon 3 in KLF4α causes the deletion of the nuclear localization signal and zinc-finger DNA binding domain, which causes KLF4α cytoplasmic sequestration in comparison to full-length KLF4, which is primarily found in the nucleus, as is expected of nuclear transcription factors. KLF4α has also been identified in normal tissues, suggesting that it is not specific to pathological contexts (15). Interestingly, there is a longer intron-retaining human KLF4 isoform, hKLF4a, identified independently by two groups from myeloid cells (17) and B-cell acute lymphoblastic leukemia samples (16). This isoform retains a 102 bp sequence between human KLF4 exons 3 and 4 that corresponds to an additional 34 amino acids in KLF4a protein. While classified as an intron-retaining isoform, the upstream regulatory elements and splicing control mechanisms for hKLF4a remain unknown. In addition, while intron-retaining KLF4 isoforms have not been reported in mice, a similar 116-bp sequence between mouse KLF4 exons 3 and 4, as the 102-bp sequence used by hKLF4a, was found. This suggests a mouse ortholog of hKLF4a isoform may exist, although the intron is not in-frame, perhaps indicating possible species-specific regulation.

**Table 2:** KLF4α and KLF4a isoforms and their regulators and functions.

KLF4 isoform	AS and domains	Cells targeted	Phenotypes	References
KLF4α	Exon 3 skipping (no C-termial Zinc fingers)	Epithelial cells of tumor or normal tissues (lung, breast, kidney, ovary, prostate)	Antagnostic to KLF4 in pancreatic cancer	D. Wei (14)
			Antagnostic to KLF4 in breast cancer	J.Ferralli (15)
			Cytoplasmic localization in prostate cancer	C Le Magnen (64)
			Both KLF4α and KLF4 promote melanoma	M Riverso (65)
KLF4α (?)	exon skipping	liver cells	Non functional exon-skipping KLF4 transcripts	Q Shen (66)
KLF4a	Intron 4 retaining (extra 34 amino acids)	B-cells	unknown	D Malik (16)
		myeloid cells	unknown	J. D. Noti (17)

While the details of KLF4 AS in different contexts are unclear, there are reports of factors relevant to the spliceosome in regulating KLF4 splicing. In hepatocellular carcinoma, splicing factor 3B subunit 4 (SF3B4), a component of the spliceosome, facilitates the generation of KLF4 exon-skipping isoforms and contributes to the malignant transformation and growth of hepatocytes (64). Physical interaction between KLF4 and SF3B4 was independently confirmed (65). In addition, DEAD-box RNA helicase 21 (DDX21) was found in the large protein complex containing RNA splicing factors and promoted the splicing of several key pro-differentiation genes, including KLF4, during a screening of glucose-binding proteins (66). The importance of DDX proteins in regulating KLF4 splicing was also shown by its physical interactions with DDX17 in hepatocellular carcinoma (67) and with DDX3X in breast cancer cell cycle progression (68). However, in these studies with DDX proteins, the nature of AS, either intron-skipping or intron-retaining, of KLF4 pre-mRNA was not known (**Table 3**). On the other hand, studies of other factors in the large complex of the spliceosome, such as small nuclear RNA (snRNA), serine/arginine-rich proteins, and many other regulatory RNA-binding proteins, have not been conducted. Additionally, at the chromatin/epigenetic level, little is known about the role of specific chromatin modification enzymes and chromatin remodeling factors that are critical to the regulation of KLF4 AS in different contexts.

**Table 3:** Regulators of KLF4 alternative splicing.

Regulator	Mechanism	References
SF3B4	Exon skipping by RNA-Seq	Q Shen (64)
DDX21	Loss of DDX21 → exon skipping by CLIP	W Miao (66)
DDX17	Binding with KLF4 pre-mRNA	Y Xue (67)
DDX3X	Binding with KLF4 pre-mRNA	E Cannizzaro (68)

→, indicates the outcome of loss of DDX21.

Despite the identification of multiple KLF4 isoforms, it remains unknown whether these variants exert divergent effects on immune reprogramming, such as promoting M2 polarization, sustaining efferocytosis, or modulating cytokine profiles in trained macrophages. Likewise, in breast cancer stem cells, differential nuclear localization of KLF4α could influence the plasticity of EMT/MET transitions. Future investigations utilizing long-read RNA sequencing, splice isoform-specific antibodies, or CRISPR-mediated isoform knockout may be necessary to dissect the role of individual KLF4 isoforms in immune cell plasticity and cancer stem cell behavior. Moreover, given that trained immunity is regulated by histone modifications (e.g., H3K4me1, H3K27ac), future studies should investigate whether isoform-specific splicing of KLF4 is modulated by chromatin state. The potential for bivalent chromatin at the KLF4 locus in stem-like immune or cancer cells is an intriguing but untested hypothesis.

## 5 KLF4 in wound healing and cancer

### 5.1 KLF4 in wound healing and tissue repair

KLF4 plays an essential role in maintaining skin barrier integrity and orchestrating wound re-epithelialization (9). For example, we previously reported a wound-healing model that suggests KLF4-expressing cells are derived from multipotent hair follicle stem cells (HFSCs), which migrate to the wound to promote healing. Upon ablation of KLF4 expression, the HFSC-enriched population was decreased, which led to a significant impairment of wound healing (69). KLF4 is also directly implicated in wound healing mechanisms through the KLF4-mediated differentiation of MDSCs into fibrocytes. Without KLF4, MDSC populations are reduced, which leads to the inability to form fibrocytes and thus causes a delay in wound healing (29). One mechanism by which KLF4 promotes wound healing through the differentiation of MDSCs is the suppression of Th17 CD4^+^ T cells. In a diabetic wound model, KLF4 activation increased MDSCs in the wound, decreasing the cytokines necessary for Th17 differentiation (70). These examples support KLF4’s role in enhancing wound healing through the modulation of inflammatory responses, supporting stem cell maintenance, and tissue regeneration.

### 5.2 KLF4 and tumor immune modulation via MDSCs

The interplay between KLF4 and myeloid cells is especially pertinent in the context of cancer, where KLF4’s role in modulating immune responses can influence tumor progression and metastasis. Depending on the context, KLF4 plays a dual role in cancer, functioning as a tumor suppressor or as an oncogene (63). KLF4 also promotes cancer development through its regulation of MDSC differentiation and function. We have previously demonstrated in a metastatic breast cancer model that KLF4 knockdown reduces granulocyte-macrophage colony-stimulating factor (GM-CSF), which is essential for MDSC development, through regulation of CXCL5, delaying tumor development. This suggests that KLF4 contributes to breast cancer tumorigenesis by regulating MDSC function (71). Likewise, knockout of KLF4 has been shown to reduce melanoma metastasis to the lung with concomitant reduction of MDSCs by reducing the development of MDSCs from fibrocytes through the regulation of fibroblast-specific protein-1 (FSP-1) (28). KLF4-driven fibrocyte differentiation also implicates this transcription factor in extracellular matrix remodeling and desmoplastic stroma formation, critical in tumor metastasis and drug resistance.

### 5.3 KLF4 in oncogenesis and cancer metabolism

KLF4 also acts as an oncogene. The overexpression of KLF4 has been implicated in the transformation of epithelium in the early development of head and neck squamous cell carcinoma (HNSCC) (72). Additionally, it is known that KLF4 negatively regulates the transcription of p53 and can, therefore, inhibit apoptosis (48). KFL4’s oncogenic potential is also linked to its metabolic regulation in breast cancer, which usually has high KLF4 expression. Moon et al. demonstrated that KLF4 binds the promoter of phosphofructokinase (PFKP) to activate glycolytic metabolism and proliferation of breast cancer cells (73). Conversely, the overexpression of KLF4 in colorectal cancer (74) and gastric cancer (75) has been shown to reduce tumorigenicity. In a cohort of patients with neuroblastoma, a rare cancer of immature nerve cells, low expression of KLF4 was associated with poor clinical outcomes. Moreover, the overexpression of KLF4 in a neuroblastoma cell line suppressed cellular proliferation by upregulating p21, a cell cycle inhibitor, and shifted the phenotype of the cell to become more epithelial-like and non-tumorigenic (76), further highlighting KLF4’s link to cellular plasticity. Given KLF4’s role in regulating glycolytic enzymes and mitochondrial integrity, its isoform-specific metabolic reprogramming may underlie both trained immunity in myeloid cells and the metabolic flexibility of cancer stem cells.

Research into KLF4’s complex role in tumorigenesis and pluripotency regulation is ongoing; however, it is clear that KLF4 has broad effects. One such study identified that KLF4 binds the promoter of human telomerase reverse transcriptase (hTERT), thereby activating telomerase, which is a hallmark of cancer. Knockdown of KLF4 in human HNSCC cell lines reduced hTERT expression and telomerase activity. Additionally, they found that in human embryonic stem cells (hESCs), KLF4 is essential for maintaining hTERT expression and a pluripotent phenotype (77). This mechanistic link to hTERT may partially explain KLF4’s role in maintaining pluripotency during iPSC reprogramming.

### 5.4 KLF4 and breast cancer stem cell plasticity

Breast cancer stem cells (BCSCs), a subpopulation of cancer cells that possess self-renewal properties and act as the seed of breast cancer, have been proposed as drivers of dormancy and relapse in breast cancer (78). Thus, it has long been thought that targeting BCSCs holds great promise in treating breast cancer recurrence (79, 80). However, recent observations of a high degree of plasticity intrinsic to BCSCs pose a serious challenge to the development of targeted therapeutic strategies (81, 82). It has been shown that BCSCs can change between mesenchymal-like (EMT-like BCSCs) and epithelial-like (MET-like BCSCs) states in a process regulated by the tumor microenvironment. Specifically targeting the molecular factors involved in a specific BCSC state may result in BCSC reversion to the alternative state and, consequently, therapy failure. By contrast, targeting factors that control BCSC plasticity may effectively eradicate this population by preventing its adaptation to fluctuating tumor microenvironments.

KLF4 plays a role in BCSCs and breast cancer metastasis. Yu et al. demonstrated that KLF4 is highly expressed in CSC-enriched breast cancer populations, and KLF4 knockdown decreased the proportion of cells with stem cell phenotypic markers and decreased the production of mammospheres. Importantly, a reduction in KLF4 results in reduced metastatic potential of breast cancer cells (83). Notably, the existence and functional roles of KLF4 isoforms in BCSCs remain uncharacterized, representing a critical research gap. KLF4 may act through miR-200/ZEB1, WNT/β-catenin, or YAP/TAZ signaling to regulate state transitions in BCSCs. Additionally, post-translational modification or isoform variation of KLF4 may alter its capacity to repress EMT-TFs like Snail or Twist.

While current studies associate full-length KLF4 with cancer stemness and tumor immune evasion, it remains unknown whether alternative isoforms such as KLF4α or KLF4a modulate these processes differently. Isoforms lacking DNA-binding domains may act as dominant-negative regulators in BCSCs or skew myeloid-derived fibrocyte differentiation in wound repair. Clarifying the spatiotemporal expression and function of KLF4 isoforms in the tumor microenvironment is essential.

## 6 Clinical and therapeutic implications

The developing field of trained immunity may have considerable implications for cancer immunotherapy. Elucidating the isoform- and context-specific roles of KLF4 in myeloid reprogramming could lead to novel therapeutic targets. β-glucan is known to induce trained immunity and has been shown to counteract immune tolerance through inhibition of immune-responsive gene 1 (IRG1) (84) and be protective against *Mycobacterium tuberculosis* challenge, which is dependent upon interleukin-1 (IL-1) signaling and associated with an expansion of hematopoietic stem cells (HSCs) and increased myelopoiesis (85). As discussed earlier, KLF4 is extrinsically linked to myelopoiesis, further highlighting its role in cellular plasticity and stemness.

Given β-glucan’s dual role in innate memory and tumor suppression (86), the KLF4 axis presents a compelling therapeutic target; further investigation into KLF4 as a target for cancer immunotherapy is warranted. In recent years, immune checkpoint inhibitors (ICIs) have become a promising cancer immunotherapy. They function by inhibiting tumor cell suppression of effector immune cells, most commonly T cells. While ICIs have been revolutionary for many patients with solid and hematological malignancies, complications of immune-related adverse events hamper their therapeutic effects (87, 88). A recent study by Xia et al. has implicated macrophage polarization to inflammatory M1 phenotype as a mediator of ICI-induced cardiac injury, and KLF4 was decreased due to miR-34a regulation after ICI treatment, suggesting that KLF4 may be a therapeutic target for reducing immunotherapy-induced cardiac injury (89). KLF4 can affect tumor immunity and responses to cancer therapies, including newly promising ICIs, by modulating macrophage phenotypes between pro- and anti-inflammatory states. As described above, KLF4 plays a key role in immune-metabolic crosstalk by regulating glycolysis and lipid metabolism during macrophage polarization, aligning metabolic state with inflammatory function (22). Its ability to repress glycolytic pathways in M2 macrophages while supporting oxidative metabolism positions KLF4 centrally in coordinating metabolic and immune programming.

In addition to its roles in cancer and trained immunity, KLF4, a Yamanaka factor, plays a central role in stem cell identity, and its expression is tightly epigenetically regulated, and it has been shown to promote the maintenance of pluripotency in iPSCs (73, 90). In the context of aging, KLF4 has been implicated in hematopoietic stem cell maintenance and may contribute to immune rejuvenation by supporting trained immunity and monocyte reprogramming (91). KLF4 expression declines with age, leading to disruption of circadian control of immune responses. Restoration of KLF4 in aged macrophages may mitigate some effects of age-related immune dysfunction (92). The mechanisms by which KLF4 exerts its effects include the modulation of epigenetic landscapes, such as DNA methylation and histone modifications, which are critical for maintaining cellular identity and plasticity (93). KLF4 has been shown to recruit CBP/p300 to promote H3K27ac (41), or to prepress transcription by recruiting HDACs, silencing inflammatory genes (42, 43). Manipulation of these mechanisms may allow for targeted epigenetic training of monocytes without systemic inflammation. In one such example, KLF4 has been implicated in the epigenetic reprogramming of monocytes in bacillus Calmette-Guérin (BCG) vaccination, which enhances cellular responsiveness to subsequent infections, as demonstrated in a controlled randomized clinical trial using attenuated yellow fever virus (94). These data highlight the potential for developing new therapeutic strategies to enhance immunity to infections and cancer by targeting KLF4 and its associated signaling and epigenetic pathways.

As our understanding of KLF4 isoforms expands, it is increasingly plausible that distinct variants may exert differential—or even opposing—effects on trained immunity, macrophage polarization, and immune tolerance. These differences could significantly influence the durability, direction, and even reversibility of therapeutic responses in cancer, infectious diseases, or inflammatory disorders. A more nuanced, isoform-specific view of KLF4 activity will be critical for refining immunotherapeutic strategies that aim to harness or reprogram myeloid cell function.

Moreover, as a better understanding of the roles of KLF4 isoforms in physiologic and pathologic contexts emerges, more targeted and potentially more effective therapies could be developed. Several therapeutic strategies can be envisioned to selectively manipulate KLF4 isoform activity in clinical settings. Small molecules or antisense oligonucleotides could be designed to enhance or silence specific KLF4 isoforms depending on the immunological context. Epigenetic modulators, such as HDAC inhibitors, may be leveraged to promote expression of full-length KLF4 in trained monocytes, thereby reinforcing durable immune memory. Targeted delivery of KLF4-modulating agents to macrophages using nanoparticle or antibody-based platforms could allow for cell-specific immunomodulation with reduced off-target effects. However, it is important to note that therapeutic manipulation of KLF4 must be approached with caution, as certain isoforms are associated with oncogenic potential (48, 63), activation of telomerase (hTERT) (77), or reprogramming of cancer stem cells (83).

Given that KLF4 plays an important role in modulating MDSC-derived fibrocytes during wound healing (29), therapeutic modulation of KLF4 isoforms may carry important implications in regenerative medicine. However, KLF4-driven fibrocyte differentiation raises concerns about unintended fibrosis and must be carefully regulated.

Furthermore, KLF4 isoform expression profiles could serve as predictive biomarkers for responsiveness to trained immunity-inducing vaccines or as indicators of susceptibility to immune-related adverse events during immune checkpoint therapy, guiding more personalized and precise interventions.

Together, these emerging insights underscore the importance of viewing KLF4 not as a singular transcription factor, but as a family of isoforms with distinct functions in immune regulation. As research advances, translating this molecular complexity into targeted therapies and even predictive diagnostics could transform how we modulate innate immune memory and myeloid plasticity across an array of pathologies.

## 7 Conclusion and future perspectives

**Figure 2:**
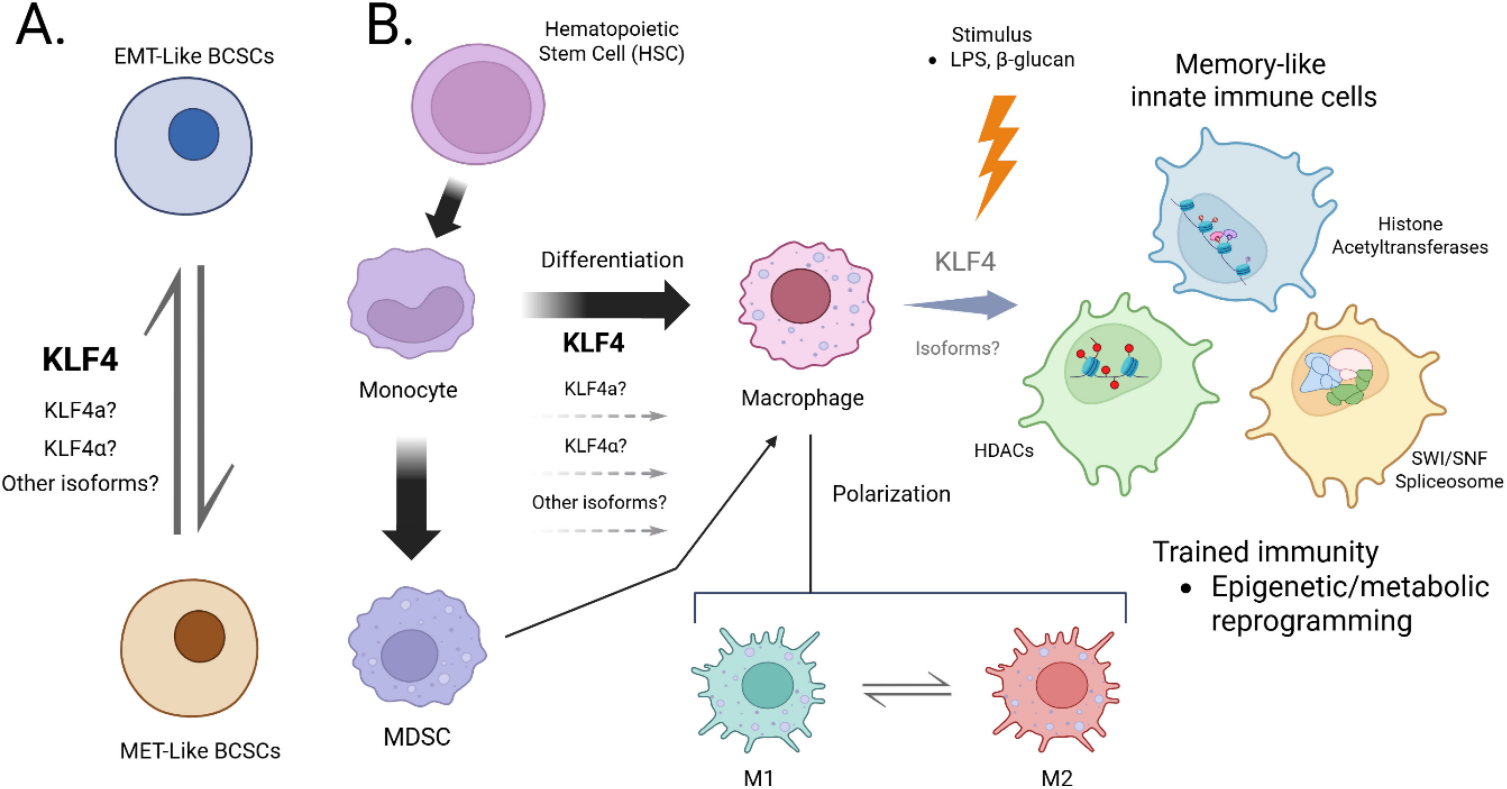
Schematic model linking KLF4 isoform diversity to the regulation of cellular plasticity in cancer and trained immunity. **(A)**. KLF4α (exon 3–deleted) may antagonize nuclear KLF4 activity in EMT-like BCSCs by cytoplasmic sequestration. **(B)**. KLF4a (intron-retaining) may participate in the transcriptional programming of monocytes/macrophages during trained immunity. Grey isoforms denote predicted variants with unknown function.

KLF4 is a complex transcription factor that plays several pivotal roles in the regulation of stem cells and myeloid cells, including their differentiation, polarization, and functional responses. KLF4 has been implicated in several mechanisms important for cellular plasticity and trained immunity. Its ability to regulate metabolic and epigenetic pathways shows that KLF4 is a key player in developing, maintaining, and suppressing immune responses. Our central hypothesis is that KLF4 plasticity, as shown by the existence of different isoforms, controls the plasticity of myeloid cells, as illustrated by different populations of MDSCs and M1/M2 macrophages, an underlying mechanism bridging cancer and trained immunity. An interesting question to consider is to what extent KLF4 is involved in the cross-talk between innate and adaptive immune responses, given KLF4’s role in reprogramming innate cells to have immune memory while controlling the differentiation of myeloid progenitors to have different levels and modes of stimulatory or suppressive activity in the adaptive immune response. In addition, given the existence of different KLF4 isoforms, representing a distinct layer of molecular plasticity, it will be critical to further study the relationship between the molecular plasticity of KLF4 and the plasticity of stem cells and immune cells (**Figure 2**), which will be essential for future KLF4-based therapeutic strategies in various inflammatory conditions and cancer. Specifically, unique KLF4 isoforms should be used in future studies when describing KLF4 function instead of only the canonical full-length KLF4. Understanding how KLF4 isoforms are generated by AS of KLF4 pre-mRNA in BCSCs and myeloid cell plasticity will uncover novel ways, either by targeting specific KLF4 isoforms or the splicing regulators controlling the dynamics of KLF4 isoforms, in treating breast cancer and modifying the memory effects of myeloid cells.

## References

[1] Netea MG Quintin J van der Meer JW. Trained immunity: a memory for innate host defense. Cell Host Microbe. (2011) 9:355–61. doi: 10.1016/j.chom.2011.04.006 21575907

[2] Bowdish DM Loffredo MS Mukhopadhyay S Mantovani A Gordon S. Macrophage receptors implicated in the “adaptive” form of innate immunity. Microbes Infect. (2007) 9:1680–7. doi: 10.1016/j.micinf.2007.09.002 18023392

[3] Netea MG Dominguez-Andres J Barreiro LB Chavakis T Divangahi M Fuchs E. Defining trained immunity and its role in health and disease. Nat Rev Immunol. (2020) 20:375–88. doi: 10.1038/s41577-020-0285-6 PMC7186935 32132681

[4] McConnell BB Yang VW. Mammalian Kruppel-like factors in health and diseases. Physiol Rev. (2010) 90:1337–81. doi: 10.1152/physrev.00058.2009 PMC2975554 20959618

[5] Ghaleb AM Yang VW. Kruppel-like factor 4 (KLF4): What we currently know. Gene. (2017) 611:27–37. doi: 10.1016/j.gene.2017.02.025 28237823 PMC5391259

[6] Wang L Shen F Stroehlein JR Wei D. Context-dependent functions of KLF4 in cancers: Could alternative splicing isoforms be the key? Cancer Lett. (2018) 438:10–6. doi: 10.1016/j.canlet.2018.09.005 30217565

[7] Monticelli S Natoli G. Transcriptional determination and functional specificity of myeloid cells: making sense of diversity. Nat Rev Immunol. (2017) 17:595–607. doi: 10.1038/nri.2017.51 28580958

[8] Feinberg MW Wara AK Cao Z Lebedeva MA Rosenbauer F Iwasaki H. The Kruppel-like factor KLF4 is a critical regulator of monocyte differentiation. EMBO J. (2007) 26:4138–48. doi: 10.1038/sj.emboj.7601824 PMC2230668 17762869

[9] Segre JA Bauer C Fuchs E. Klf4 is a transcription factor required for establishing the barrier function of the skin. Nat Genet. (1999) 22:356–60. doi: 10.1038/11926 10431239

[10] Takahashi K Yamanaka S. Induction of pluripotent stem cells from mouse embryonic and adult fibroblast cultures by defined factors. Cell. (2006) 126:663–76. doi: 10.1016/j.cell.2006.07.024 16904174

[11] Christ A Gunther P Lauterbach MAR Duewell P Biswas D Pelka K. Western diet triggers NLRP3-dependent innate immune reprogramming. Cell. (2018) 172:162–75 e14. doi: 10.1016/j.cell.2017.12.013 29328911 PMC6324559

[12] Chakraborty S Singh A Wang L Wang X Sanborn MA Ye Z. Trained immunity of alveolar macrophages enhances injury resolution via KLF4-MERTK-mediated efferocytosis. J Exp Med. (2023) 220. doi: 10.1084/jem.20221388 PMC10450795 37615937

[13] Wang N Hu Y Wang Z. Regulation of alternative splicing: Functional interplay with epigenetic modifications and its implication to cancer. Wiley Interdiscip Rev RNA. (2023):e1815. 37697868 10.1002/wrna.1815

[14] Wei D Wang L Kanai M Jia Z Le X Li Q. KLF4alpha up-regulation promotes cell cycle progression and reduces survival time of patients with pancreatic cancer. Gastroenterology. (2010) 139:2135–45. doi: 10.1053/j.gastro.2010.08.022 PMC3245983 20727893

[15] Ferralli J Chiquet-Ehrismann R Degen M. KLF4alpha stimulates breast cancer cell proliferation by acting as a KLF4 antagonist. Oncotarget. (2016) 7:45608–21. doi: 10.18632/oncotarget.10058 PMC5216746 27323810

[16] Malik D Kaul D Chauhan N Marwaha RK. miR-2909-mediated regulation of KLF4: a novel molecular mechanism for differentiating between B-cell and T-cell pediatric acute lymphoblastic leukemias. Mol Cancer. (2014) 13:175. doi: 10.1186/1476-4598-13-175 25037230 PMC4112645

[17] Noti JD Johnson AK Dillon JD. The leukocyte integrin gene CD11d is repressed by gut-enriched Kruppel-like factor 4 in myeloid cells. J Biol Chem. (2005) 280:3449–57. doi: 10.1074/jbc.M412627200 15561714

[18] Wan J Su Y Song Q Tung B Oyinlade O Liu S. Methylated cis-regulatory elements mediate KLF4-dependent gene transactivation and cell migration. Elife. (2017) 6. doi: 10.7554/eLife.20068 PMC5466421 28553926

[19] Ben-Meir K Twaik N Baniyash M. Plasticity and biological diversity of myeloid derived suppressor cells. Curr Opin Immunol. (2018) 51:154–61. doi: 10.1016/j.coi.2018.03.015 29614426

[20] Schouppe E De Baetselier P Van Ginderachter JA Sarukhan A. Instruction of myeloid cells by the tumor microenvironment: Open questions on the dynamics and plasticity of different tumor-associated myeloid cell populations. Oncoimmunology. (2012) 1:1135–45. doi: 10.4161/onci.21566 PMC3494626 23170260

[21] Liang Y Zhao J Dai T Li X Chen L He Z. A review of KLF4 and inflammatory disease: Current status and future perspective. Pharmacol Res. (2024) 207:107345. doi: 10.1016/j.phrs.2024.107345 39134187

[22] Liao X Sharma N Kapadia F Zhou G Lu Y Hong H. Kruppel-like factor 4 regulates macrophage polarization. J Clin Invest. (2011) 121:2736–49. doi: 10.1172/JCI45444 PMC3223832 21670502

[23] Tang RZ Zhu JJ Yang FF Zhang YP Xie SA Liu YF. DNA methyltransferase 1 and Kruppel-like factor 4 axis regulates macrophage inflammation and atherosclerosis. J Mol Cell Cardiol. (2019) 128:11–24. doi: 10.1016/j.yjmcc.2019.01.009 30659837

[24] Ye Q Luo F Yan T. Transcription factor KLF4 regulated STAT1 to promote M1 polarization of macrophages in rheumatoid arthritis. Aging (Albany NY). (2022) 14:5669–80. doi: 10.18632/aging.204128 PMC9365561 35748767

[25] Wang K Xiong J Lu Y Wang L Tian T. SENP1-KLF4 signalling regulates LPS-induced macrophage M1 polarization. FEBS J. (2023) 290:209–24. doi: 10.1111/febs.v290.1 35942612

[26] Bashore AC Xue C Kim E Yan H Zhu LY Pan H. Monocyte single-cell multimodal profiling in cardiovascular disease risk states. Circ Res. (2024) 135:685–700. doi: 10.1161/CIRCRESAHA.124.324457 39105287 PMC11430373

[27] Condamine T Ramachandran I Youn JI Gabrilovich DI. Regulation of tumor metastasis by myeloid-derived suppressor cells. Annu Rev Med. (2015) 66:97–110. doi: 10.1146/annurev-med-051013-052304 25341012 PMC4324727

[28] Shi Y Ou L Han S Li M Pena MM Pena EA. Deficiency of Kruppel-like factor KLF4 in myeloid-derived suppressor cells inhibits tumor pulmonary metastasis in mice accompanied by decreased fibrocytes. Oncogenesis. (2014) 3:e129. doi: 10.1038/oncsis.2014.44 25417726 PMC4259966

[29] Ou L Shi Y Dong W Liu C Schmidt TJ Nagarkatti P. Kruppel-like factor KLF4 facilitates cutaneous wound healing by promoting fibrocyte generation from myeloid-derived suppressor cells. J Invest Dermatol. (2015) 135:1425–34. doi: 10.1038/jid.2015.3 PMC4402119 25581502

[30] Nimpong JA Gebregziabher W Singh UP Nagarkatti P Nagarkatti M Hodge J. Deficiency of KLF4 compromises the lung function in an acute mouse model of allergic asthma. Biochem Biophys Res Commun. (2017) 493:598–603. doi: 10.1016/j.bbrc.2017.08.146 28867182 PMC5636673

[31] Xu L Zhou C Liang Y Fan T Zhang F Chen X. Epigenetic modifications in the accumulation and function of myeloid-derived suppressor cells. Front Immunol. (2022) 13:1016870. doi: 10.3389/fimmu.2022.1016870 36439186 PMC9691837

[32] Karpurapu M Ranjan R Deng J Chung S Lee YG Xiao L. Kruppel like factor 4 promoter undergoes active demethylation during monocyte/macrophage differentiation. PloS One. (2014) 9:e93362. doi: 10.1371/journal.pone.0093362 24695324 PMC3973678

[33] Stavast CJ Leenen PJM Erkeland SJ. The interplay between critical transcription factors and microRNAs in the control of normal and Malignant myelopoiesis. Cancer Lett. (2018) 427:28–37. doi: 10.1016/j.canlet.2018.04.010 29673909

[34] Tang D Kang R Coyne CB Zeh HJ Lotze MT. PAMPs and DAMPs: signal 0s that spur autophagy and immunity. Immunol Rev. (2012) 249:158–75. doi: 10.1111/j.1600-065X.2012.01146.x PMC3662247 22889221

[35] Mitroulis I Ruppova K Wang B Chen LS Grzybek M Grinenko T. Modulation of myelopoiesis progenitors is an integral component of trained immunity. Cell. (2018) 172:147–61 e12. doi: 10.1016/j.cell.2017.11.034 29328910 PMC5766828

[36] Kleinnijenhuis J Quintin J Preijers F Joosten LA Ifrim DC Saeed S. Bacille Calmette-Guerin induces NOD2-dependent nonspecific protection from reinfection via epigenetic reprogramming of monocytes. Proc Natl Acad Sci U S A. (2012) 109:17537–42. doi: 10.1073/pnas.1202870109 PMC3491454 22988082

[37] Vetvicka V. Glucan-immunostimulant, adjuvant, potential drug. World J Clin Oncol. (2011) 2:115–9. doi: 10.5306/wjco.v2.i2.115 PMC3095473 21603320

[38] Lee SM Lee JW Cho J Choi S Kim I Pack CG. Yeast-derived particulate beta-glucan induced angiogenesis via regulating PI3K/Src and ERK1/2 signaling pathway. Int J Biol Macromol. (2024) 269:131884. doi: 10.1016/j.ijbiomac.2024.131884 38685541

[39] Fanucchi S Dominguez-Andres J Joosten LAB Netea MG Mhlanga MM. The intersection of epigenetics and metabolism in trained immunity. Immunity. (2021) 54:32–43. doi: 10.1016/j.immuni.2020.10.011 33220235

[40] Moorlag S Folkman L Ter Horst R Krausgruber T Barreca D Schuster LC. Multi-omics analysis of innate and adaptive responses to BCG vaccination reveals epigenetic cell states that predict trained immunity. Immunity. (2024) 57:171–87 e14. doi: 10.1016/j.immuni.2023.12.005 38198850

[41] Evans PM Zhang W Chen X Yang J Bhakat KK Liu C. Kruppel-like factor 4 is acetylated by p300 and regulates gene transcription via modulation of histone acetylation. J Biol Chem. (2007) 282:33994–4002. doi: 10.1074/jbc.M701847200 17908689

[42] Yoshida T Gan Q Owens GK. Kruppel-like factor 4, Elk-1, and histone deacetylases cooperatively suppress smooth muscle cell differentiation markers in response to oxidized phospholipids. Am J Physiol Cell Physiol. (2008) 295:C1175–82. doi: 10.1152/ajpcell.00288.2008 PMC2584997 18768922

[43] Salmon M Gomez D Greene E Shankman L Owens GK. Cooperative binding of KLF4, pELK-1, and HDAC2 to a G/C repressor element in the SM22alpha promoter mediates transcriptional silencing during SMC phenotypic switching *in vivo* . Circ Res. (2012) 111:685–96. doi: 10.1161/CIRCRESAHA.112.269811 PMC3517884 22811558

[44] Moonen JR Chappell J Shi M Shinohara T Li D Mumbach MR. KLF4 recruits SWI/SNF to increase chromatin accessibility and reprogram the endothelial enhancer landscape under laminar shear stress. Nat Commun. (2022) 13:4941. doi: 10.1038/s41467-022-32566-9 35999210 PMC9399231

[45] Bao X Tang J Lopez-Pajares V Tao S Qu K Crabtree GR. ACTL6a enforces the epidermal progenitor state by suppressing SWI/SNF-dependent induction of KLF4. Cell Stem Cell. (2013) 12:193–203. doi: 10.1016/j.stem.2012.12.014 23395444 PMC3661004

[46] Chen K Long Q Xing G Wang T Wu Y Li L. Heterochromatin loosening by the Oct4 linker region facilitates Klf4 binding and iPSC reprogramming. EMBO J. (2020) 39:e99165. doi: 10.15252/embj.201899165 31571238 PMC6939195

[47] Frazzi R. KLF4 is an epigenetically modulated, context-dependent tumor suppressor. Front Cell Dev Biol. (2024) 12:1392391. doi: 10.3389/fcell.2024.1392391 39135777 PMC11317372

[48] Rowland BD Bernards R Peeper DS. The KLF4 tumour suppressor is a transcriptional repressor of p53 that acts as a context-dependent oncogene. Nat Cell Biol. (2005) 7:1074–82. doi: 10.1038/ncb1314 16244670

[49] Pradhan K Yi Z Geng S Li L. Development of exhausted memory monocytes and underlying mechanisms. Front Immunol. (2021) 12:778830. doi: 10.3389/fimmu.2021.778830 34777396 PMC8583871

[50] Cooper MA Elliott JM Keyel PA Yang L Carrero JA Yokoyama WM. Cytokine-induced memory-like natural killer cells. Proc Natl Acad Sci U S A. (2009) 106:1915–9. doi: 10.1073/pnas.0813192106 PMC2644138 19181844

[51] Sun JC Beilke JN Lanier LL. Adaptive immune features of natural killer cells. Nature. (2009) 457:557–61. doi: 10.1038/nature07665 PMC2674434 19136945

[52] Wang X Peng H Tian Z. Innate lymphoid cell memory. Cell Mol Immunol. (2019) 16:423–9. doi: 10.1038/s41423-019-0212-6 PMC6474199 30796350

[53] Cheng M Li J Song J Song H Chen Y Tang H. RORalpha is required for expansion and memory maintenance of ILC1s via a lymph node-liver axis. Cell Rep. (2024) 43:113786. doi: 10.1016/j.celrep.2024.113786 38363684

[54] Yamada T Park CS Mamonkin M Lacorazza HD. Transcription factor ELF4 controls the proliferation and homing of CD8+ T cells via the Kruppel-like factors KLF4 and KLF2. Nat Immunol. (2009) 10:618–26. doi: 10.1038/ni.1730 PMC2774797 19412182

[55] Imbriano C Belluti S. Histone marks-dependent effect on alternative splicing: new perspectives for targeted splicing modulation in cancer? Int J Mol Sci. (2022) 23. doi: 10.3390/ijms23158304 PMC9368390 35955433

[56] Malakar P Shukla S Mondal M Kar RK Siddiqui JA. The nexus of long noncoding RNAs, splicing factors, alternative splicing and their modulations. RNA Biol. (2024) 21:1–20. doi: 10.1080/15476286.2023.2286099 PMC10761143 38017665

[57] Bernard A Boidot R Vegran F. Alternative splicing in cancer and immune cells. Cancers (Basel). (2022) 14. doi: 10.3390/cancers14071726 PMC8996879 35406498

[58] He Z He J Xie K. KLF4 transcription factor in tumorigenesis. Cell Death Discov. (2023) 9:118. doi: 10.1038/s41420-023-01416-y 37031197 PMC10082813

[59] Cheng Z Zou X Jin Y Gao S Lv J Li B. The role of KLF(4) in alzheimer’s disease. Front Cell Neurosci. (2018) 12:325. doi: 10.3389/fncel.2018.00325 30297986 PMC6160590

[60] Aguirre M Escobar M Forero Amezquita S Cubillos D Rincon C Vanegas P. Application of the yamanaka transcription factors oct4, sox2, klf4, and c-myc from the laboratory to the clinic. Genes (Basel). (2023) 14. doi: 10.3390/genes14091697 PMC10531188 37761837

[61] Godmann M Kromberg I Mayer J Behr R. The mouse Kruppel-like Factor 4 (Klf4) gene: four functional polyadenylation sites which are used in a cell-specific manner as revealed by testicular transcript analysis and multiple processed pseudogenes. Gene. (2005) 361:149–56. doi: 10.1016/j.gene.2005.07.025 16185820

[62] Yang Y Xiong J Wang J Ruan Y Zhang J Tian Y. Novel alternative splicing variants of Klf4 display different capacities for self-renewal and pluripotency in mouse embryonic stem cells. Biochem Biophys Res Commun. (2020) 532:377–84. doi: 10.1016/j.bbrc.2020.08.054 32883521

[63] Rowland BD Peeper DS. KLF4, p21 and context-dependent opposing forces in cancer. Nat Rev Cancer. (2006) 6:11–23. doi: 10.1038/nrc1780 16372018

[64] Shen Q Eun JW Lee K Kim HS Yang HD Kim SY. Barrier to autointegration factor 1, procollagen-lysine, 2-oxoglutarate 5-dioxygenase 3, and splicing factor 3b subunit 4 as early-stage cancer decision markers and drivers of hepatocellular carcinoma. Hepatology. (2018) 67:1360–77. doi: 10.1002/hep.29606 29059470

[65] Liu Z Li W Pang Y Zhou Z Liu S Cheng K. SF3B4 is regulated by microRNA-133b and promotes cell proliferation and metastasis in hepatocellular carcinoma. EBioMedicine. (2018) 38:57–68. doi: 10.1016/j.ebiom.2018.10.067 30391496 PMC6306498

[66] Miao W Porter DF Lopez-Pajares V Siprashvili Z Meyers RM Bai Y. Glucose dissociates DDX21 dimers to regulate mRNA splicing and tissue differentiation. Cell. (2023) 186:80–97 e26. doi: 10.1016/j.cell.2022.12.004 36608661 PMC10171372

[67] Xue Y Jia X Li C Zhang K Li L Wu J. DDX17 promotes hepatocellular carcinoma progression via inhibiting Klf4 transcriptional activity. Cell Death Dis. (2019) 10:814. doi: 10.1038/s41419-019-2044-9 31653828 PMC6814716

[68] Cannizzaro E Bannister AJ Han N Alendar A Kouzarides T. DDX3X RNA helicase affects breast cancer cell cycle progression by regulating expression of KLF4. FEBS Lett. (2018) 592:2308–22. doi: 10.1002/feb2.2018.592.issue-13 PMC6100109 29782654

[69] Li J Zheng H Wang J Yu F Morris RJ Wang TC. Expression of Kruppel-like factor KLF4 in mouse hair follicle stem cells contributes to cutaneous wound healing. PloS One. (2012) 7:e39663. doi: 10.1371/journal.pone.0039663 22745808 PMC3379995

[70] Yang X Mathis BJ Huang Y Li W Shi Y. KLF4 promotes diabetic chronic wound healing by suppressing th17 cell differentiation in an MDSC-dependent manner. J Diabetes Res. (2021) 2021:7945117. doi: 10.1155/2021/7945117 34568499 PMC8457977

[71] Yu F Shi Y Wang J Li J Fan D Ai W. Deficiency of Kruppel-like factor KLF4 in mammary tumor cells inhibits tumor growth and pulmonary metastasis and is accompanied by compromised recruitment of myeloid-derived suppressor cells. Int J Cancer. (2013) 133:2872–83. doi: 10.1002/ijc.v133.12 PMC3796127 23737434

[72] Foster KW Ren S Louro ID Lobo-Ruppert SM McKie-Bell P Grizzle W. Oncogene expression cloning by retroviral transduction of adenovirus E1A-immortalized rat kidney RK3E cells: transformation of a host with epithelial features by c-MYC and the zinc finger protein GKLF. Cell Growth Differ. (1999) 10:423–34. 10392904

[73] Moon JS Kim HE Koh E Park SH Jin WJ Park BW. Kruppel-like factor 4 (KLF4) activates the transcription of the gene for the platelet isoform of phosphofructokinase (PFKP) in breast cancer. J Biol Chem. (2011) 286:23808–16. doi: 10.1074/jbc.M111.236737 PMC3129162 21586797

[74] Dang DT Chen X Feng J Torbenson M Dang LH Yang VW. Overexpression of Kruppel-like factor 4 in the human colon cancer cell line RKO leads to reduced tumorigenecity. Oncogene. (2003) 22:3424–30. doi: 10.1038/sj.onc.1206413 PMC2275074 12776194

[75] Wei D Gong W Kanai M Schlunk C Wang L Yao JC. Drastic down-regulation of Kruppel-like factor 4 expression is critical in human gastric cancer development and progression. Cancer Res. (2005) 65:2746–54. doi: 10.1158/0008-5472.CAN-04-3619 15805274

[76] Shum CK Lau ST Tsoi LL Chan LK Yam JW Ohira M. Kruppel-like factor 4 (KLF4) suppresses neuroblastoma cell growth and determines non-tumorigenic lineage differentiation. Oncogene. (2013) 32:4086–99. doi: 10.1038/onc.2012.437 23045286

[77] Wong CW Hou PS Tseng SF Chien CL Wu KJ Chen HF. Kruppel-like transcription factor 4 contributes to maintenance of telomerase activity in stem cells. Stem Cells. (2010) 28:1510–7. doi: 10.1002/stem.477 20629177

[78] De Angelis ML Francescangeli F Zeuner A. Breast cancer stem cells as drivers of tumor chemoresistance, dormancy and relapse: new challenges and therapeutic opportunities. Cancers (Basel). (2019) 11. doi: 10.3390/cancers11101569 PMC6826533 31619007

[79] Das PK Rakib MA Khanam JA Pillai S Islam F. Novel therapeutics against breast cancer stem cells by targeting surface markers and signaling pathways. Curr Stem Cell Res Ther. (2019) 14:669–82. doi: 10.2174/1574888X14666190628104721 31808385

[80] Dittmer J. Breast cancer stem cells: Features, key drivers and treatment options. Semin Cancer Biol. (2018) 53:59–74. doi: 10.1016/j.semcancer.2018.07.007 30059727

[81] Liu S Cong Y Wang D Sun Y Deng L Liu Y. Breast cancer stem cells transition between epithelial and mesenchymal states reflective of their normal counterparts. Stem Cell Rep. (2014) 2:78–91. doi: 10.1016/j.stemcr.2013.11.009 PMC3916760 24511467

[82] Sousa B Ribeiro AS Paredes J. Heterogeneity and plasticity of breast cancer stem cells. Adv Exp Med Biol. (2019) 1139:83–103. 31134496 10.1007/978-3-030-14366-4_5

[83] Yu F Li J Chen H Fu J Ray S Huang S. Kruppel-like factor 4 (KLF4) is required for maintenance of breast cancer stem cells and for cell migration and invasion. Oncogene. (2011) 30:2161–72. doi: 10.1038/onc.2010.591 PMC3088782 21242971

[84] Dominguez-Andres J Novakovic B Li Y Scicluna BP Gresnigt MS Arts RJW. The itaconate pathway is a central regulatory node linking innate immune tolerance and trained immunity. Cell Metab. (2019) 29:211–20 e5. doi: 10.1016/j.cmet.2018.09.003 30293776

[85] Moorlag S Khan N Novakovic B Kaufmann E Jansen T van Crevel R. beta-Glucan Induces Protective Trained Immunity against Mycobacterium tuberculosis Infection: A Key Role for IL-1. Cell Rep. (2020) 31:107634. doi: 10.1016/j.celrep.2020.107634 32433977 PMC7242907

[86] Murphy EJ Rezoagli E Major I Rowan NJ Laffey JG. beta-glucan metabolic and immunomodulatory properties and potential for clinical application. J Fungi (Basel). (2020) 6. doi: 10.3390/jof6040356 PMC7770584 33322069

[87] Abu-Sbeih H Ali FS Naqash AR Owen DH Patel S Otterson GA. Resumption of immune checkpoint inhibitor therapy after immune-mediated colitis. J Clin Oncol. (2019) 37:2738–45. doi: 10.1200/JCO.19.00320 PMC6800279 31163011

[88] Bockstahler M Fischer A Goetzke CC Neumaier HL Sauter M Kespohl M. Heart-specific immune responses in an animal model of autoimmune-related myocarditis mitigated by an immunoproteasome inhibitor and genetic ablation. Circulation. (2020) 141:1885–902. doi: 10.1161/CIRCULATIONAHA.119.043171 32160764

[89] Xia W Zou C Chen H Xie C Hou M. Immune checkpoint inhibitor induces cardiac injury through polarizing macrophages via modulating microRNA-34a/Kruppel-like factor 4 signaling. Cell Death Dis. (2020) 11:575. doi: 10.1038/s41419-020-02778-2 32709878 PMC7382486

[90] Doeser MC Scholer HR Wu G. Reduction of fibrosis and scar formation by partial reprogramming *in vivo* . Stem Cells. (2018) 36:1216–25. doi: 10.1002/stem.2842 29761584

[91] Blacher E Tsai C Litichevskiy L Shipony Z Iweka CA Schneider KM. Aging disrupts circadian gene regulation and function in macrophages. Nat Immunol. (2022) 23:229–36. doi: 10.1038/s41590-021-01083-0 PMC9704320 34949832

[92] Yang JH Petty CA Dixon-McDougall T Lopez MV Tyshkovskiy A Maybury-Lewis S. Chemically induced reprogramming to reverse cellular aging. Aging (Albany NY). (2023) 15:5966–89. doi: 10.18632/aging.204896 PMC10373966 37437248

[93] Tetens AR Martin AM Arnold A Novak OV Idrizi A Tryggvadottir R. DNA methylation landscapes in DIPG reveal methylome variability that can be modified pharmacologically. Neurooncol Adv. (2024) 6:vdae023. doi: 10.1093/noajnl/vdae023 38468866 PMC10926944

[94] Arts RJW Moorlag S Novakovic B Li Y Wang SY Oosting M. BCG Vaccination Protects against Experimental Viral Infection in Humans through the Induction of Cytokines Associated with Trained Immunity. Cell Host Microbe. (2018) 23:89–100 e5. doi: 10.1016/j.chom.2017.12.010 29324233

